# Use of primary care physiotherapy and its associations with clinical and socioeconomic outcomes in musculoskeletal disorders: a cohort study

**DOI:** 10.1080/02813432.2026.2660328

**Published:** 2026-05-05

**Authors:** Olav Amundsen, Tron Anders Moger, Jon Helgheim Holte, Silje Bjørnsen Haavaag, Line K. Bragstad, Ragnhild Hellesø, Trond Tjerbo, Nina Køpke Vøllestad

**Affiliations:** ^a^ Department of Public Health Science and Interdisciplinary Health Science, Institute of Health and Society, University of Oslo, Oslo, Norway; ^b^ Department of Health Management and Health Economics, Institute of Health and Society, University of Oslo, Oslo, Norway; ^c^ Department of Rehabilitation Science and Health, Faculty of Health Sciences, Oslo Metropolitan University, Oslo, Norway

**Keywords:** Physiotherapy, musculoskeletal diseases, prognosis, health planning, treatment outcome

## Abstract

**Objectives:**

To assess how physiotherapy contact frequency relates to clinical outcomes in patients with musculoskeletal disorders across prognostic groups, and how outcomes are associated with future healthcare costs and disability pension.

**Design and setting:**

Cohort study linking clinical data from the FYSIOPRIM primary care physiotherapy cohort (12-month follow-up) with national registry data on healthcare use, costs, and socioeconomic factors.

**Participants:**

1475 patients were included in FYSIOPRIM at baseline, 671 provided data on all covariates and 12-month outcomes and were included in the analysis.

**Method:**

Comparing low and high physiotherapy use during a 12 month follow-up period (High use: Median or higher (9+ contacts)). Patients were categorized into good, medium, and poor clinical prognosis based on seven baseline clinical factors.

**Main outcome measures:**

Global Perceived Effect (GPE) at 12 months, high healthcare costs (≥95th percentile two years post-FYSIOPRIM), and disability pension (1–3 years post-FYSIOPRIM).

**Results:**

High physiotherapy use was associated with improvement in GPE in patients with poor prognosis (OR 4.04, 95%CI 1.56–10.50) but not for good or medium prognosis. Improvement on GPE was associated with lower odds of high future healthcare costs (OR 0.27, 95%CI 0.13–0.58) and disability pension (OR 0.06, 95%CI 0.01–0.53).

**Conclusions:**

Patients with poor prognosis may benefit from more physiotherapy contacts, while those with better prognosis may require fewer contacts. Tailoring treatment intensity to prognosis may support more efficient resource allocation and support long-term societal benefits through reduced healthcare costs and disability risk.

## Introduction

Musculoskeletal disorders (MSDs) are among the most common reasons for patients seeking help from the healthcare system, with approximately one-third of the Norwegian population consulting healthcare services for MSDs annually [1,2]. MSDs are the leading cause of years lived with disability and can result in large individual consequences with activity-limitations, disability and reduced participation in work and social life [3,4].

Many patients with MSDs have a favorable prognosis and recover well with simple self-management advice and minimal help from healthcare services, while other patients have more complex presentations with poorer prognoses, which may require more intensive management [5]. Physiotherapy is a commonly used service in primary care management for MSDs and provides care that has been shown to be cost-effective with few side-effects [6,7]. Although many patients report positive outcomes, it is unclear how much of the effect of physiotherapy treatment is attributed to natural recovery, regression to the mean, contextual effects or specific effects related to the care they receive [8]. A recent systematic review showed that a greater number of physiotherapy contacts was not associated with better clinical outcomes [9]. This raises questions about whether there is any benefit to patients with MSDs having a high number of physiotherapy contacts, as compared to a lower use, and whether this differs between patients with different clinical prognoses [8,9]. In this study, clinical outcome refers to patient-reported improvement after physiotherapy, while clinical prognoses reflect an estimated prognosis based on a set of baseline clinical factors previously shown to have prognostic value for MSDs.

Musculoskeletal disorders (MSDs) have a large impact on societal resource use through high health expenditures and are the most common work-related health problem in Europe [10]. Previous research has shown that demographic and socioeconomic factors, as well as clinical factors, comorbidities and health status are associated with healthcare costs and work participation [11,12]. Early and guideline-adherent physiotherapy is associated with lower future healthcare costs, and short-term clinical improvement after physiotherapy treatment is associated with lower use of additional healthcare services [13,14]. However, there is limited research examining whether self-reported improvement achieved in primary care is associated with future long-term outcomes that are important in a societal perspective.

This study aims to assess how the frequency of physiotherapy use is associated with self-reported improvement for patients with different clinical prognoses in primary care physiotherapy. Furthermore, it examines the relationship between self-reported improvement and future healthcare costs and disability pensions. Addressing these questions will provide evidence to guide clinical decision-making, optimize resource allocation and inform policies aimed at improving both clinical and societal outcomes for patients with MSDs.

## Methods

### Study design, data sources, and setting

This cohort study combines clinical data from the Research Program for Physiotherapy in Primary Health Care (FYSIOPRIM) with national registries covering healthcare use, demographic and socioeconomic factors [15]. FYSIOPRIM was a Norwegian longitudinal study of primary care physiotherapy, that included patients across nine municipalities and 36 clinics, with 12-month follow-up [16]. Baseline clinical data came from FYSIOPRIM, demographic and socioeconomic data from Statistics Norway, and work participation, sick leave and disability pensions from Statistics Norway’s historical events database (FD-Trygd). Healthcare use and costs were extracted from The Control and reimbursement of healthcare claims (KUHR) database for primary care, and the Norwegian Patient Registry (NPR) for specialist care. We captured all MSD-related healthcare use up to 12 months, and MSD-related healthcare costs up to three years. The Norwegian Cause of Death Registry was used to exclude patients that died during the follow-up period. This study adheres to the Strengthening the Reporting of Observational Studies in Epidemiology (STROBE) guideline for observational studies [17].

### Inclusion criteria

We included patients that had been included in the FYSIOPRIM-database from 2015 to 2020. The original database consists of 4071 registrations. We included patients registered with a diagnosis related to a musculoskeletal condition in FYSIOPRIM, excluding postoperative patients, who could be identified in KUHR and NPR. We excluded patients that were registered in FYSIOPRIM with a private-sector physiotherapist and patients that did not have any registered one-on-one physiotherapy contacts in KUHR the following year after FYSIOPRIM inclusion. Additionally, we only included patients 18 years or older and excluded patients who died within three years after baseline. We included both patients with new and ongoing disorders. In FYSIOPRIM, patients report at baseline whether they present with a new or ongoing problem. In our population, 86% were registered with a new problem. We chose not to exclude patients based on this criterion, as this reflects clinical practice, where most patients present with new problems while a smaller proportion have received treatment prior to consulting the physiotherapist.

### Index date and follow-up period

Patients were registered with an index date based on the date they were included in the FYSIOPRIM-database. This index date was used to create a follow-up period, where we registered all healthcare contacts for the following year after the individual index date.

### Healthcare use

All physiotherapy contacts in the one-year follow-up period were registered. We included all physiotherapy contacts regardless of diagnosis in KUHR, as the patient had been given a musculoskeletal diagnosis in FYSIOPRIM. Only contacts with reimbursement codes indicating a one-on-one consultation were included. We categorized physiotherapy contacts in the 12-month period into low and high (high use defined as median or higher, median was 9 contacts) as this has been used in previous research [18]. This variable was used to assess the association between low and high frequency of physiotherapy contacts and clinical outcomes.

General practitioner (GP) and specialist care use were included as covariates in the analysis. All contacts with a GP indicating a face-to-face consultation with an MSD-diagnosis in the follow-up period were registered. For GP use we defined the 90th percentile (three contacts) as a cut-off between low and high use. We registered specialist care use by including all outpatient contacts and inpatient stays with an MSD-diagnosis. This includes contact with any healthcare personnel in specialist care. Musculoskeletal conditions include all ICD-10 codes chapter M, and the code G55. Patients registered with the specific codes related to infections (M00, M01, M02), malignant disease (M86), and inflammatory rheumatic disease (M05-M08, M10, M11, M13, M30-M36, M45, M46) were excluded, as this study focuses on symptom-based MSDs. We also included injuries to the musculoskeletal system from codes within the chapter ‘Injury, poisoning and certain other consequences of external causes’ related to MSD, codes S32-34, S40-S99 and T08- T13, excluding codes related to superficial injuries and wounds (Sx0 and Sx1). Specialist care use was dichotomized to use/no use.

### Clinical prognosis

We used seven clinical factors, previously shown to have prognostic value for MSDs, to categorize patients into good, medium and poor prognosis [19,20]. This follows the logic from established prognostic tools, such as the STarT MSK Tool, where several factors are combined to produce a stratification tool [21]. These clinical factors were registered in FYSIOPRIM and used: PSFS, pain intensity last week (0-10), HSCL10) pain duration, fear avoidance (1 item from Tampa Scale of Kinesiophobia), vitality item from 15D, and EQ-5d utility score [19,22–27]. Each factor was categorized as either low or high risk, with high risk indicating greater severity. We used the 25th and 75th percentiles as cutoff for low and high, except for variables with well-established cutoffs (HSCL10 at 1.85) or categorical values (pain duration of 12 months or more). Patients that filled out five or more clinical factors were included. Patients that scored high-risk on less than 25% of the clinical factors were classified as good prognosis, patients with high-risk scores on more than 25% and up to 50% were classified as medium prognosis, and patients that scored high-risk on more than 50% were classified as poor prognosis. This definition was used as we were interested in categorizing patients with different clinical prognoses based on multiple clinical factors into account, rather than using clinical factors as individual covariates.

### Covariates

Age, gender, education level and immigration background were included from Statistics Norway. Work status was registered as employed/self-employed or not registered as employed. We registered sick leave status, defined as being registered with a sick leave at baseline, or within 31 days after baseline. Being registered on disability pension at baseline was also included as a covariate.

Diagnosis was registered by the treating physiotherapist in FYSIOPRIM in the initial consultation. This was registered with an ICPC-2 code and with the opportunity to provide further detail in free text. In this study, we categorized diagnosis to osteoarthritis (registered with osteoarthritis as diagnosis or registered as knee or hip pain and above 50 years of age), spinal pain, shoulder pain, knee pain (excluding those specified with osteoarthritis and patients above 50 years of age), widespread pain/fibromyalgia, fractures/injuries and other MSDs.

Comorbidities were registered using a previously adapted comorbidity index from GP-diagnoses based on selected ICPC-2 diagnoses, which have been validated to be used as an adjustment variable in epidemiological research in primary care databases [27]. The comorbidity index was dichotomized into 0/1 or 2+ comorbidities.

### Outcomes

We used Global Perceived Effect (GPE) as the clinical outcome in this study. GPE is a 7-item scale ranging from ‘very much improved’ to ‘very much worse’. This was dichotomized into a binary variable where ‘very much improved’ and ‘much improved’ were classified as improved, and answers ranging from ‘a little improvement’ to ‘very much worse’ were classified as not improved. GPE is a commonly used outcome measure for MSDs and allows the patient to assess whether they have experienced improvement based on their own assessment of what is important to themselves [28].

Future high-cost healthcare service use was used as an outcome measure. High-cost healthcare service use was defined as patients with MSD-related healthcare costs at or above the 95th percentile during the two years after the follow-up period. All costs in this study are based on the reimbursement costs and activity-based funding registered in KUHR and NPR. This means that our data represents the cost for the public healthcare system to reimburse the actual services provided. The patients’ out-of-pocket costs or costs related to capitation from the municipality or block grants to hospitals is not included, as we did not have access to these data.

Future disability pension was used as an outcome measure. We assessed disability pension after the FYSIOPRIM follow-up period, until the end of 2019, which was the last registration of disability pensions in our data. We have used whether the patient is registered with any disability pension at any time from after the one-year FYSIOPRIM follow-up period until the end of 2019 as an outcome. Disability pension is determined by a lack of work function, and not on diagnosis alone, and there is no registration of diagnosis in the data. This means that we can determine whether the patient receives disability pension, but not if it is directly related to their MSDs.

### Statistical analysis

We used descriptive statistics to summarize demographic and socioeconomic factors, general health, work participation and welfare use, clinical factors, diagnosis, healthcare use and outcomes. These statistics were reported for the full population and stratified by clinical prognosis. We conducted a comparison of nonresponders and responders on all baseline characteristics, healthcare use and socioeconomic outcomes, using T-test for variables with normal distribution, Wilcoxon rank-sum test (Mann–Whitney *U* test) for variables with non-normal distribution, and chi-square test for categorical variables.

We used univariate and multivariate logistic regression analysis to analyze associations between physiotherapy use and clinical outcome, and between clinical outcome and future high costs and disability pension. Multivariate models controlled for other healthcare use (GP and specialist care) demographic, socioeconomic, general health, sick leave and disability pension status, diagnosis, and clinical prognostic factors previously shown to be prognostic for MSDs [19]. Associations between physiotherapy use and clinical outcomes were stratified based on clinical prognosis, based on the hypothesis that clinical prognosis influences healthcare needs. To assess the direction of the association and the risk of reverse causality between physiotherapy use and clinical outcome, we examined associations between early (0–3 months) and late (3–12 months) physiotherapy use and clinical outcomes at 12 months, as well as between clinical outcomes at 3 months and late physiotherapy use. Covariate selection was guided by directed acyclic graphs (DAGs) [29].

The analysis using future high-cost healthcare service use as an outcome could only include patients that were included 2018 or earlier, to allow us to capture healthcare costs for two years after the follow-up period. The analysis using disability pension as an outcome measure included patients registered in work the year before baseline, 63 years or younger, not registered with prior disability pension and included in FYSIOPRIM in 2017 or earlier.

Due to the use of previously registered data, no formal sample size calculation was performed. For the outcomes future high healthcare costs and disability pension, the number of events was low relative to the number of covariates, resulting in fewer than ten events per predictor variable (EPV) [28]. As the aim was to assess associations while adjusting for relevant confounders rather than to develop a prediction model, all covariates were retained in the analyses. Sensitivity analyses using backward elimination yielded similar results, indicating that the findings were robust despite the low EPV.

To assess the robustness of our findings, we conducted several sensitivity analyses: testing alternative definitions of clinical prognosis (using higher and lower cutoff values, and by including factors as individual covariates); analyses using healthcare use as continuous variables instead of categorized; multiple imputation using chained equations to estimate missing 12-month GPE values based on available 3-month GPE data and relevant covariates; and analyses including both individual and group consultations (for patients with at least one one-on-one consultation). Including group consultations led to no association between low or high use and clinical outcome for the full sample (OR 1.42, 95%CI 0.97–2.09), but a higher odds ratio for the poor prognosis group (OR 5.52, 95%CI 1.93–15.80) as compared to the primary analysis. Across all other sensitivity analyses, the results remained consistent with the findings presented in the manuscript.

## Results

**Figure 1.**
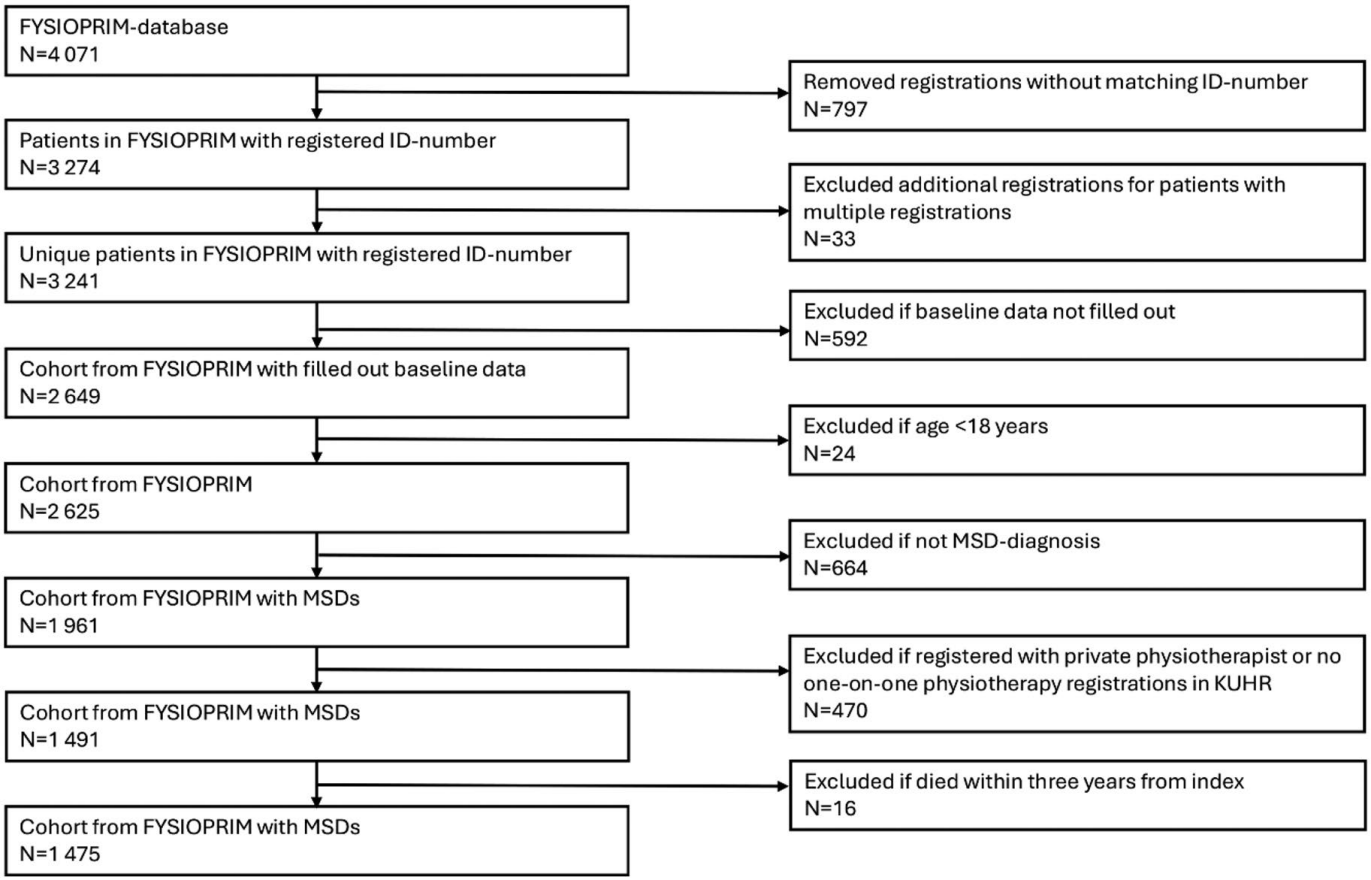
Flowchart.

Out of 1475 patients included at baseline, 700 patients completed clinical outcome measure at 12 months, and 671 of these provided data on all covariates and were used in the analyses. The sample selection process is illustrated in Figure 1.

Table 1 presents baseline characteristics for the full cohort and stratified by clinical prognosis. Patients with poor prognosis were younger, had less education, more often had an immigrant background, were on sick leave or received disability pension. The patients with poor prognosis more frequently had widespread pain or fibromyalgia as diagnosis at inclusion and had the highest healthcare service use. The median number of physiotherapy contacts were 9 (IQR 15) contacts for the full sample. Patients with good prognosis had a median of 6 (IQR 11) contacts, medium prognosis a median of 10 (IQR 16) and poor prognosis a median of 13 (IQR 19) contacts. The good prognosis group had a median of 1 (IQR 2) GP contacts, the medium prognosis had a median of 1 (IQR 3), and the poor prognosis group had 2 (IQR 4) GP contacts. The proportion of patients with at least one MSD-related hospital contact was 23%, 30% and 39%, respectively, for the patients with good, medium and poor prognosis. There was a relatively similar use of chiropractor between the groups, with 8.7%, 10.7% and 10.7% of patients in good, medium and poor prognoses groups that had any contact with a chiropractor.

**Table 1.** Baseline patient characteristics for all included patients, presented stratified by clinical prognosis.

	All *N* = 1475*	Good prognosis *N* = 577	Medium prognosis *N* = 533	Poor prognosis *N* = 327
**Demographic factors**				
Age	50.6 (17.0)	52.7 (17.3)	50.2 (16.7)	47.6 (16.7)
Sex, female	72.0%	69.3%	73.9%	73.7%
**Socioeconomic factors**				
Education, more than 13 years	51.8%	56.0%	50.9%	45.7%
Immigration background	10.2%	9.0%	9.0%	14.1%
**Work participation and welfare use**				
Employed or self-employed	70.8%	71.2%	72.1%	67.8%
Sick leave at, or within 1 month, from baseline	23.2%	18.0%	25.9%	28.1%
Disability pension before baseline	7.4%	3.8%	8.3%	12.2%
**General health**				
Comorbidity, two or more	4.9%	4.3%	4.3%	7.0%
Body Mass Index	26.3 (4.8)	25.8 (4.5)	26.3 (4.6)	27.3 (5.5)
**Clinical factors**				
PSFS	3.6 (2.3)	4.4 (2.2)	3.5 (2.4)	2.6 (2.0)
Pain intensity last week	4.5 (2.2)	3.3 (1.6)	4.6 (2.0)	6.5 (1.8)
Pain duration, 12 months or more	48.6%	29.4%	54.2%	73.3%
HSCL10**	1.66 (0.51)	1.36 (0.29)	1.64 (0.41)	2.19 (0.54)
Fear avoidance	3.2 (2.9)	1.9 (2.1)	3.7 (3.0)	4.8 (2.9)
Vitality 15D	2.2 (1.0)	1.7 (0.6)	2.2 (0.9)	3.2 (0.9)
EQ5d utility	0.62 (0.19)	0.73 (0.09)	.62 (0.17)	0.43 (0.20)
**Diagnosis**				
Osteoarthritis	27.4%	28.4%	29.3%	22.3%
Spinal	26.9%	24.2%	21.0%	26.9%
Shoulder	13.2%	14.4%	11.8%	13.5%
Knee	8.6%	7.8%	10.9%	6.1%
Widespread/Fibromyalgia	8.5%	5.2%	6.4%	17.7%
Fracture/injuries	4.2%	6.1%	3.6%	2.1%
Other MSDs	14.5%	13.9%	17.1%	11.3%

*38 patients were not categorized to prognosis due to missing variables

**Hopkins Symptom Checklist 10.

Presented as mean (SD) or percentage.

Out of the 671 patients that completed 12-month outcomes and provided data on all covariates, 49% of the patients with poor prognosis reported improvement on global perceived effect (GPE) at 12 months, while 77% and 68% reported improvement in the groups with good and medium prognosis, respectively. Out of 624 patients included 2018 or earlier and completed 12-month outcomes and provided data on all covariates, the proportion of patients classified with future high-costs service use were 3.5%, 4.9% and 8.3%, respectively, for the groups with good, medium and poor clinical prognosis. Out of the 248 patients registered in work the year before baseline, 63 years or younger, not registered with prior disability pension and included in FYSIOPRIM in 2017 or earlier, 5.2% received disability pension in the years after the follow-up period. This proportion were 0.1%, 3.8% and 18.2%, respectively, in the groups with good, medium and poor prognosis.

Comparison of nonresponders and responders showed that nonresponders were significantly younger (responders: mean age 52.6 (SD 16.6), nonresponders: mean age 48.3 (SD 17.2)), had less education (responders: 54.8% with more than 13 years, nonresponders: 49.2%), higher proportion with immigrant background (responders: 8%, nonresponders: 12%), had worse scores on HSCL10 (responders: mean 1.61 (SD 0.48), nonresponders: 1.71 (SD 0.54)), Vitality 15D (responders: mean 2.1 (SD 0.9), nonresponders: mean 2.3 (SD 1.0)) and EQ5d utility (responders: mean 0.64 (SD 0.18), nonresponders: mean 0.60 (SD 0.20)). Responders had more GP contacts (responders: median 1 (IQR 3), nonresponders: median 1 (IQR 2)), and there was no difference in physiotherapy, hospital or chiropractor use. There were no differences in socioeconomic outcomes.

### Associations between physiotherapy use and clinical outcome

**Figure 2.**
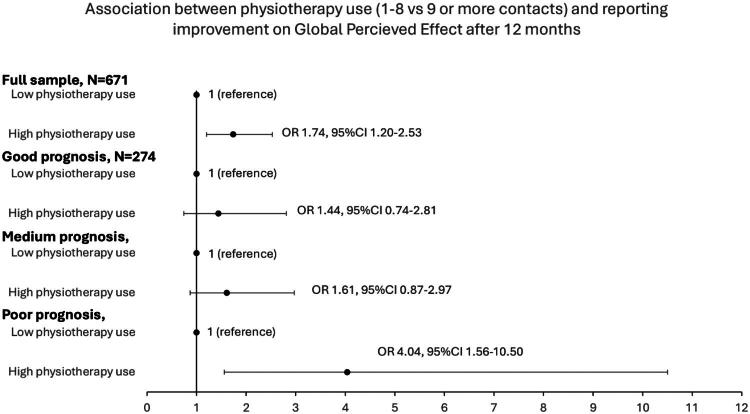
Associations between low and high physiotherapy use and reporting improvement on Global Perceived Effect after 12 months for the full sample and stratified by clinical prognosis. Analyzed with multivariate logistic regression analysis.

For the full sample, patients with high physiotherapy use were more likely to report improvement on GPE compared to patients with lower use (OR 1.74, 95%CI 1.20–2.53) when controlling for other healthcare use, demographic, socioeconomic, general health factors, sick leave and disability pension status, diagnosis, and clinical prognostic factors, as shown in Figure 2. When stratified by clinical prognosis, the results showed that patients with poor prognosis were four times more likely (OR 4.04, 95%CI 1.56–10.50) to report a good clinical outcome if they had a high use of physiotherapy compared to a lower use, while there was no association for patients with good or medium prognoses. Full multivariate logistic regression models are included in supplementary.

When analyzing data on physiotherapy use and clinical outcome at 3 months and 12 months to indicate the direction of the association, the results support that higher physiotherapy use led to improved outcomes, and not the opposite. We found a significant association between high use at 0 to 3 months and reporting improvement on GPE at 12 months (OR 1.51, 95%CI 1.05–2.15), and between high use at 3 to 12 months and reporting improvement at 12 months (OR 1.71, 95%CI 1.03–2.84). Reporting improvement at 3 months was associated with lower odds of high physiotherapy use at 3 to 12 months (OR 0.58, 95% CI 0.37–0.90).

### Associations between clinical outcomes and socioeconomic outcomes

**Figure 3.**
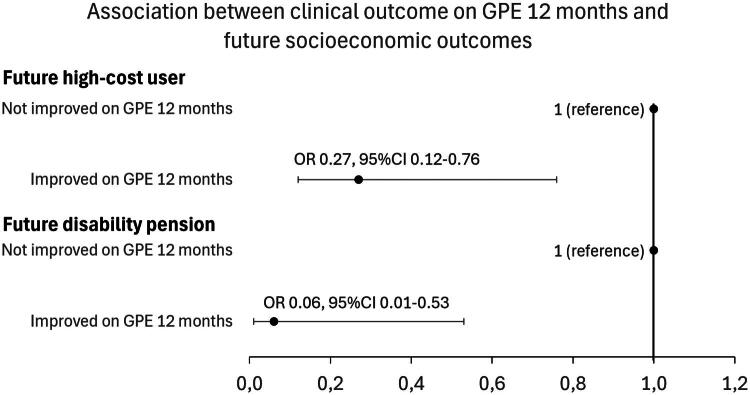
Associations between reporting improvement on GPE after 12 months and risk of being future high-cost user (costs above the 95th percentile the two years after the follow-up period) (*N* = 624) and future disability pension (registered with disability pension at least 365 days after baseline and before 2020) (*N* = 248). Analyzed with multivariate logistic regression analysis.

The analysis with future high-cost service (*N* = 624), and analysis with future disability pension as outcome (*N* = 248) showed that reporting improvement on GPE at 12 months was associated with lower risk of having a high-cost service use the two years following the FYSIOPRIM follow-up period, and lower risk of future disability pension, when controlling for healthcare use, demographic, socioeconomic, general health, diagnosis, and clinical prognostic factors (Figure 3). The full multivariate logistic regression analyses including all covariates are included in supplementary.

## Discussion

### Comparison to previous studies

Higher use of physiotherapy was associated with improvement on GPE after 12 months. When stratifying the analysis based on prognosis, we found that patients with poor prognosis were four times more likely to report a positive outcome if they received a high number of physiotherapy contacts, as compared to a lower number of contacts. This contrasts with previous studies, suggesting that low use of physiotherapy may be equally effective as more comprehensive physiotherapy treatment programs for MSDs [9]. One possible explanation could be the inclusion of clinical prognosis as a variable, which may allow us to detect associations between frequency of use and self-reported improvement that are not apparent in studies that does not control for clinical prognoses. Another explanation could be that our study reflects real-world clinical practice where treatment is provided by the treating physiotherapists discretion, unlike prior studies that use standardized interventions. Our study looks at differences in frequency of contacts for real-life application of physiotherapy treatment, where treatment can be tailored to the individual, and several factors influence both treatment and frequency of contacts. This may produce different results compared to studies where interventions are based on a standardized protocol. Differences in design, patient populations and inclusion settings may also contribute to the differences in our findings compared to prior studies [29–32].

We also found that achieving a good clinical outcome after 12 months was associated with lower odds of having a future high-cost service use and reduced risk of receiving disability pension. Similar to our findings, a prior study has shown that improvement on clinical outcomes at 4 weeks after their initial physiotherapy treatment was associated with lower self-reported healthcare use at 6 and 12 months [14]. These findings support that the effective management of MSDs in primary care could lead to both individual improvements and have a broader positive societal benefit.

### Implications

The findings in our study may have important implications for prioritization in clinical practice. The association between physiotherapy use in primary care and clinical outcome varies by clinical prognoses, suggesting that patients with poor prognosis may benefit from more physiotherapy contacts, while patients with good prognosis could require fewer contacts. Although there is increasing focus on overtreatment of MSDs and promotion of self-management approaches, our findings show that certain patient groups may benefit from a higher frequency of contacts in primary care physiotherapy and should be prioritized for this [33]. These findings indicate that clinical prognosis can be used to allocate the limited resources in primary care more effectively.

Our findings also have implications in a policy perspective. Due to the high prevalence and large variation in care for MSDs, there is a growing interest in clinical care pathways for MSDs [34]. Our findings suggest that patients with similar diagnoses have differing care needs, supporting an individualized treatment approach based on clinical prognosis, rather than standardized treatment based on diagnosis. When planning and designing future MSD-care, it is important to acknowledge that there is no one-size-fits-all solution, and patients with similar diagnoses may require different help from healthcare services to achieve positive outcomes.

This study suggests that treatment for patients with MSDs in primary care can improve patient outcomes, and that this is associated with reduced healthcare costs and remaining in the workforce. This indicates that the positive clinical outcome the patient achieves in primary care reduces the need of more expensive healthcare services and support work participation. The costs related to MSDs represents a significant economic and social burden, but costs for primary care services are negligible compared to specialist care and lost productivity [6,10,11]. Primary healthcare services improve health outcomes, health system efficiency and health equity, which can lead to substantial economic benefits for healthcare systems and societies [35]. Our findings support that strengthening primary care services for patients with MSDs could lead to societal cost savings, and that effective care in primary care may contribute to a sustainable healthcare system. These findings are based on a Norwegian context, but we believe that the findings of our study are generalizable to other countries with similar healthcare organizations and structures.

Our study also has important research applications. Register-based data can provide large datasets, but our study shows the importance of clinical data to understand healthcare use and outcomes for MSDs. To expand on the current knowledge and the findings from our study, it would be beneficial to collect clinical data on a larger population from different care settings that could be matched with register-data. This could add further detail and information on care pathways, healthcare use, and the individual and societal outcomes from use of different healthcare services.

### Study limitations

Our study includes only patients who sought physiotherapy to manage their MSD. This means that our findings are valid for patients going to primary care physiotherapy, but not for the broader population of patients with MSDs. A previous study showed that patients using physiotherapy were older, had slightly higher income and employment rates, had more sick leave and lower proportion with immigrant background compared to all patients with new MSDs. Among patients with more intensive physiotherapy use, there were also higher rates of disability pension, lower education and slightly more comorbidity [6]. Additionally, primary care physiotherapy has been shown to be predicted by higher baseline pain intensity and disability, as well as sick leave status [18]. This indicates that patients going to physiotherapy have worse health status, more pain and disability and more problems remaining in work compared to the general population with MSDs, and that the population in this study may not be representative for the broader population of patients with MSDs.

Out of 1475 patients, 700 completed 12-month outcomes, indicating a 50% nonresponse in the FYSIOPRIM-database, raising concerns about attrition bias [36]. Attrition bias is a common issue in cohort studies, although it is unclear whether it leads to further bias than what is already present at baseline [36,37]. Nonresponders were younger, had less education, a higher proportion had immigrant background, and they had worse baseline scores for psychosocial distress and health-related quality of life. We found no differences in socioeconomic outcomes between responders and nonresponders, suggesting that non-response was not outcome-dependent.

We chose to create a prognostic variable that classified patients into good, medium or poor prognosis based on multiple clinical factors, rather than to include the clinical factors individually. This allowed us to assess whether the association between frequency of physiotherapy use and self-reported improvement differed between patients with different prognosis. Ideally, we would have used an established and validated prognostic tool such as the STarT MSK Tool or the Örebro Musculoskeletal Pain Screening Questionnaire, but this was not possible with our data [21,38].

We dichotomized certain continuous variables, such as healthcare use, to improve the clinical interpretability. The categorization makes the variables intuitively easy to understand, and we considered that the relationship between these variables and the outcome was likely nonlinear, where each unit increase may not have the same effect on the outcome. Therefore, we considered this approach to be appropriate in our study. However, categorization of continuous factors can increase the risk of false positive findings, as arbitrary categorization can bias findings toward a significant result and can lead to loss of information and statistical power [39]. To account for this limitation, we conducted sensitivity analyses including healthcare use variables as continuous factors, and these results were consistent with the findings presented in the manuscript.

Diagnoses for MSDs in primary care is challenging due to high symptom overlap and patients commonly have multiple pain sites [40]. We used ICPC-2 codes and free text diagnosis from FYSIOPRIM to increase the diagnostic validity compared to only using register-based data. Following the same diagnosis over time in registries is challenging due to varying coding practices, systems and software between settings and clinics [41]. We believe that including patients based on data from a clinical database and registering contacts coded with any musculoskeletal diagnosis in the registries is a reasonable approach.

We included only one-on-one consultations in the primary analyses. As our aim was to compare patients with low or high healthcare use, we considered it most appropriate to focus on one-on-one consultations, which represent the most common form of physiotherapy contacts. However, excluding group consultations may underestimate healthcare use in certain patient populations. Group consultations may be used as a strategy to manage patients expected to require many consultations, and excluding these contacts may not fully capture healthcare use in subgroups of patients. Sensitivity analyses including group consultations showed no association between low or high use and clinical outcome for the full sample, while the association for the poor prognosis group remained significant.

## Conclusions

Our findings show that patients with poor clinical prognosis may benefit from more physiotherapy contacts, while patients with good and medium prognoses can be managed with fewer contacts. We also found that a good clinical outcome in primary care physiotherapy was associated with lower odds of high future healthcare costs in the subsequent two years, and lower odds of receiving disability pension one to three years after follow-up. This could be understood as the societal benefit of patients achieving a good clinical outcome in primary care physiotherapy. This study contributes to the understanding of physiotherapy use and outcomes for patients with MSDs, and how this differs for patients with different prognoses. It also shows the importance of having access to clinical data in addition to register-data to understand healthcare use and outcomes for patients with MSDs. A future research recommendation would be to collect clinical data from a broader population and different care settings, to expand on the current knowledge base and findings from this study.

## Supplementary Material

Supplemental Material
