# Supplementary material for: Use of primary care physiotherapy and its associations with clinical and socioeconomic outcomes in musculoskeletal disorders: a cohort study
Source: Scand J Prim Health Care. 2026 May 5;44(1):2660328. doi: 10.1080/02813432.2026.2660328 (PMC13148086; doi:10.1080/02813432.2026.2660328)
Supplement: Supplemental Material [file IPRI_A_2660328_SM7762.zip › Art3_supplementary.docx]

**Supplementary:**

**Supplementary 1: Full logistic regression analysis for results used in manuscript.**

Table 1: Logistic regression – Odds ratio for reporting improvement on GPE n=671

|  | Univariate  OR (95% CI) | Multivariate  OR (95% CI) |
| --- | --- | --- |
| Physiotherapy contacts, median or higher (9+) | 1.24 (.90-1.70) | **1.74 (1.20-2.53)** |
| GP contacts, 90^th^ percentile or higher (5+) | **.52 (.33-.81)** | **.51 (.30-.86)** |
| Any hospital contact | **.59 (.42-.82)** | **.61 (.41-.92)** |
| Any chiropractor contact | .72 (.42-1.26) | **.53 (.29-.98)** |
| Age | .99 (.98-1.00) | 1.00 (.99-1.02) |
| Gender, female | .87 (.61-1.25) | .99 (.67-1.58) |
| Education, more than 13 years | 1.03 (.75-1.42) | .78 (.54-1.12) |
| Immigration background | 1.21 (.66-2.21) | 1.22 (.63-2.37) |
| Sick leave at, or within 1 month, from baseline | 1.39 (.95-2.04) | 2.10 (1.31-3.37) |
| Disability pension before baseline | **.50 (.27-.92)** | .72 (.36-1.46) |
| Comorbidity, 1 or more | **.62 (.43-.88)** | .74 (.34-1.59) |
| Body Mass Index | **.97 (.94-1.00)** | .97 (.93-1.00) |
| Clinical prognosis  Good  Medium  Poor | Ref  **.60 (.42-.88)**  **.30 (.20-.47)** | Ref  **.60 (.40-.91)**  **.31 (.19-.52)** |
| Diagnosis  Osteoarthritis  Spinal  Shoulder  Knee  Widespread/Fibromyalgia  Fracture/injuries  Other MSDs | Ref  .88 (.58-1.34)  2.12 (1.21-3.70)  2.60 (1.24-5.44)  .91 (.49-1.68)  **3.03 (1.00-9.16)**  1.33 (.80-2.24) | Ref  .64 (.38-1.07)  1.41 (.76-2.64)  2.10 (.85-5.20)  .77 (.35-1.42)  1.55 (.48-4.96)  1.02 (.54-1.96) |

Table 2: Multivariate logistic regression – Odds ratio reporting improvement on GPE, stratified by clinical prognosis

|  | Good: N=274  Multivariate  OR (95% CI) | Medium N=263  Multivariate  OR (95% CI) | Poor: N=132  Multivariate  OR (95% CI) |
| --- | --- | --- | --- |
| Physiotherapy contacts, median or higher (9+) | 1.44 (.74-2.81) | 1.61 (.87-2.97) | **4.04 (1.56-10.50)** |
| GP contacts, 90^th^ percentile or higher (5+) | .35 (.12-1.05) | **.32 (.13-.76)** | .82 (.27-2.42) |
| Any hospital contact | .86 (.39-1.86) | .79 (.40-1.55) | .41 (.15-1.05) |
| Any chiropractor contact | **.22 (.08-0.60)** | 1.59 (.54-4.65) | .24 (.03-1.75) |
| Age | 1.01 (.99-1.04) | .99 (.97-1.01) | 1.02 (.99-1.05) |
| Gender, female | 1.29 (.64-2.60) | 1.10 (.55-2.21) | .73 (.26-2.06) |
| Education, more than 13 years | .62 (.32-1.21) | .80 (.44-1.44) | 1.11 (.50-2.65) |
| Immigration background | 2.49 (.67-9.23) | 2.57 (.65-10.21) | .23 (.05-1.18) |
| Sick leave at, or within 1 month, from baseline | **5.29 (1.17-23.85)** | 2.03 (.93-4.41) | **3.26 (1.13-9.40)** |
| Disability pension before baseline | .19 (.02-2.34) | .99 (.32-3.09) | .90 (.28-2.88) |
| Comorbidity, 2 or more | .59 (.12-3.03) | .62 (.19-2.05) | .86 (.14-4.94) |
| Body Mass Index | 1.03 (.96-1.11) | **.92 (.85-.97)** | .97 (.89-1.05) |
| Diagnosis  Osteoarthritis  Spinal  Shoulder  Knee  Widespread/Fibromyalgia  Fracture/injuries  Other MSDs | Ref  2.66 (1.05-6.74)  2.54 (.92-6.99)  **8.06 (1.19-54.24)**  2.17 (.49-9.65)  4.17 (.29-7.46)  **4.41 (1.26-15.41)** | Ref  **.41 (.17-.97)**  2.28 (.67-7.77)  1.14 (.33-3.89)  .36 (.11-1.23)  .56 (.08-4.09)  .41 (.15-1.07) | Ref  **.16 (.04-.54)**  .54 (.14-2.11)  4.63 (.15-145.65)  .54 (.13-2.22)  Omitted  1.46 (.26-8.25) |

Table 3: Associations to having a high-cost service use the two years after the follow-up period in FYSIOPRIM. high-cost service use was defined as costs above the 95^th^ percentile. Analysis only includes patients that were included in FYSIOPRIM in 2015-2018 N=600

|  | Univariate  OR (95% CI) | Multivariate  OR (95% CI) |
| --- | --- | --- |
| Global Perceived Effect, improved | **.27 (.13-.58)** | **.27 (.12-.76)** |
| Physiotherapy contacts, median or higher (9+) | 1.25 (.73-2.13) | 1.32 (.56-3.09) |
| GP contacts, 90^th^ percentile or higher (5+) | 1.40 (.69-2.81) | .52 (.15-1.89) |
| Any hospital contact | **2.21 (1.30-3.76)** | 1.78 (.76-3.53) |
| Any chiropractor contact | 1.29 (.58-2.90) | 2.66 (.68-10.51) |
| Age | **1.05 (1.03-1.07)** | **1.07 (1.02-1.11)** |
| Gender, female | 1.27 (.68-2.34) | .92 (.36-2.35) |
| Education, more than 13 years | .83 (.49-1.41) | 1.19 (.52-2.69) |
| Immigration background | .48 (.15-1.56) | Omitted |
| Sick leave at, or within 1 month, from baseline | **.22 (.08-.63)** | .43 (.09-2.12) |
| Disability pension before baseline | **2.58 (1.23-5.43)** | .99 (.24-4.09) |
| Comorbidity, 2 or more | 1.88 (.73-4.88) | .33 (.04-3.02) |
| Body Mass Index | 1.03 (.97-1.08) | .95 (.86-1.05) |
| Clinical prognosis  Good  Medium  Poor | Ref  1.54 (.83-2.87)  1.47 (.72-3.01) | Ref  1.74 (.64-4.71)  2.93 (.96-8.96) |
| Diagnosis  Osteoarthritis  Spinal  Shoulder  Knee  Widespread/Fibromyalgia  Fracture/injuries  Other MSDs | Ref  **.35 (.17-.70)**  **.23 (.08-.65)**  **.08 (.01-.62)**  .43 (.16-1.12)  Omitted  **.10 (.02-.41)** | Ref  **.24 (.06-.89)**  .55 (.13-2.22)  Omitted  .54 (.11-2.78)  Omitted  .29 (.03-2.61) |

Table 4: Associations to future disability pension, registered at least 365 days after baseline. Includes patients registered in work the year before baseline, age 63 years or younger and not registered with prior disability pension N=319

|  | Univariate  OR (95% CI) | Multivariate  OR (95% CI) |
| --- | --- | --- |
| Global Perceived Effect, improved | **.15 (.06-.41)** | **.06 (.01-.53)** |
| Physiotherapy contacts, median or higher (8+) | 1.89 (.98-3.67) | .80 (.14-4.53) |
| GP contacts, 90^th^ percentile or higher (5+) | 3.58 (1.79-7.18) | 2.98 (.30-29.34) |
| Any hospital contact | 1.22 (.59-2.50) | .65 (.12-3.56) |
| Any chiropractor contact |  | .34 (.02-6.10) |
| Age | **1.08 (1.04-1.12)** | **1.17 (1.02-1.33)** |
| Sex, female | **2.75 (1.13-6.67)** | 2.52 (.40-15.80) |
| Education, more than 13 years | **.43 (.23-.84)** | .17 (.03-1.02) |
| Immigration background | .39 (.09-1.64) | Omitted |
| Comorbidity, 1 or more | 1.49 (.70-3.17) | Omitted |
| Body Mass Index | **1.10 (1.04-1.16)** | 1.07 (.93-1.21) |
| Clinical prognosis  Good  Medium  Poor | Ref  **6.60 (1.93-22.63)**  **13.24 (3.79-46.25)** | Ref  1.83 (.12-27.78)  **19.67 (1.27-304.56)** |
| Diagnosis  Osteoarthritis  Spinal  Shoulder  Knee  Widespread/Fibromyalgia  Fracture/injuries  Other MSDs | Ref  1.00 (.38-2.64)  .59 (.17-2.10)  Omitted (no events)  **2.74 (.92-8.14)**  2.09 (.56-7.75)  .50 (.15-1.63) | Ref  3.57 (.41-31.32)  1.07 (.05-21.65)  Omitted  2.39 (.06-96.43)  20.39 (.70-586.97)  3.28 (.09-117.36) |
