# Supplementary material for: Use of primary care physiotherapy and its associations with clinical and socioeconomic outcomes in musculoskeletal disorders: a cohort study
Source: Scand J Prim Health Care. 2026 May 5;44(1):2660328. doi: 10.1080/02813432.2026.2660328 (PMC13148086; doi:10.1080/02813432.2026.2660328)
Supplement: Supplemental Material [file IPRI_A_2660328_SM7762.zip › Art3_supplementary.pdf]

Supplementary:

Supplementary 1: Full logistic regression analysis for results used in manuscript.

Table 1: Logistic regression – Odds ratio for reporting improvement on GPE n=671

	Univariate<br>OR (95% CI)	Multivariate<br>OR (95% CI)
Physiotherapy contacts, median or higher (9+)	1.24 (.90-1.70)	**1.74 (1.20-2.53)**
GP contacts, 90th percentile or higher (5+)	**.52 (.33-.81)**	**.51 (.30-.86)**
Any hospital contact	**.59 (.42-.82)**	**.61 (.41-.92)**
Any chiropractor contact	.72 (.42-1.26)	**.53 (.29-.98)**
Age	.99 (.98-1.00)	1.00 (.99-1.02)
Gender, female	.87 (.61-1.25)	.99 (.67-1.58)
Education, more than 13 years	1.03 (.75-1.42)	.78 (.54-1.12)
Immigration background	1.21 (.66-2.21)	1.22 (.63-2.37)
Sick leave at, or within 1 month, from baseline	1.39 (.95-2.04)	2.10 (1.31-3.37)
Disability pension before baseline	**.50 (.27-.92)**	.72 (.36-1.46)
Comorbidity, 1 or more	**.62 (.43-.88)**	.74 (.34-1.59)
Body Mass Index	**.97 (.94-1.00)**	.97 (.93-1.00)
Clinical prognosis		
Good	Ref	Ref
Medium	**.60 (.42-.88)**	**.60 (.40-.91)**
Poor	**.30 (.20-.47)**	**.31 (.19-.52)**
Diagnosis		
Osteoarthritis	Ref	Ref
Spinal	.88 (.58-1.34)	.64 (.38-1.07)
Shoulder	2.12 (1.21-3.70)	1.41 (.76-2.64)
Knee	2.60 (1.24-5.44)	2.10 (.85-5.20)
Widespread/Fibromyalgia	.91 (.49-1.68)	.77 (.35-1.42)
Fracture/injuries	**3.03 (1.00-9.16)**	1.55 (.48-4.96)
Other MSDs	1.33 (.80-2.24)	1.02 (.54-1.96)

Table 2: Multivariate logistic regression – Odds ratio reporting improvement on GPE, stratified by clinical prognosis

	Good: N=274<br>Multivariate<br>OR (95% CI)	Medium N=263<br>Multivariate<br>OR (95% CI)	Poor: N=132<br>Multivariate<br>OR (95% CI)
Physiotherapy contacts, median or higher (9+)	1.44 (.74-2.81)	1.61 (.87-2.97)	**4.04 (1.56-10.50)**
GP contacts, 90th percentile or higher (5+)	.35 (.12-1.05)	**.32 (.13-.76)**	.82 (.27-2.42)
Any hospital contact	.86 (.39-1.86)	.79 (.40-1.55)	.41 (.15-1.05)
Any chiropractor contact	**.22 (.08-0.60)**	1.59 (.54-4.65)	.24 (.03-1.75)
Age	1.01 (.99-1.04)	.99 (.97-1.01)	1.02 (.99-1.05)
Gender, female	1.29 (.64-2.60)	1.10 (.55-2.21)	.73 (.26-2.06)

Education, more than 13 years	.62 (.32-1.21)	.80 (.44-1.44)	1.11 (.50-2.65)
Immigration background	2.49 (.67-9.23)	2.57 (.65-10.21)	.23 (.05-1.18)
Sick leave at, or within 1 month, from baseline	**5.29 (1.17-23.85)**	2.03 (.93-4.41)	**3.26 (1.13-9.40)**
Disability pension before baseline	.19 (.02-2.34)	.99 (.32-3.09)	.90 (.28-2.88)
Comorbidity, 2 or more	.59 (.12-3.03)	.62 (.19-2.05)	.86 (.14-4.94)
Body Mass Index	1.03 (.96-1.11)	**.92 (.85-.97)**	.97 (.89-1.05)
Diagnosis			
Osteoarthritis	Ref	Ref	Ref
Spinal	2.66 (1.05-6.74)	**.41 (.17-.97)**	**.16 (.04-.54)**
Shoulder	2.54 (.92-6.99)	2.28 (.67-7.77)	.54 (.14-2.11)
Knee	**8.06 (1.19-54.24)**	1.14 (.33-3.89)	4.63 (.15-145.65)
Widespread/Fibromyalgia	2.17 (.49-9.65)	.36 (.11-1.23)	.54 (.13-2.22)
Fracture/injuries	4.17 (.29-7.46)	.56 (.08-4.09)	Omitted
Other MSDs	**4.41 (1.26-15.41)**	.41 (.15-1.07)	1.46 (.26-8.25)

Table 3: Associations to having a high-cost service use the two years after the follow-up period in FYSIOPRIM. high-cost service use was defined as costs above the 95th percentile. Analysis only includes patients that were included in FYSIOPRIM in 2015-2018 N=600

	Univariate OR (95% CI)	Multivariate OR (95% CI)
Global Perceived Effect, improved	**.27 (.13-.58)**	**.27 (.12-.76)**
Physiotherapy contacts, median or higher (9+)	1.25 (.73-2.13)	1.32 (.56-3.09)
GP contacts, 90th percentile or higher (5+)	1.40 (.69-2.81)	.52 (.15-1.89)
Any hospital contact	**2.21 (1.30-3.76)**	1.78 (.76-3.53)
Any chiropractor contact	1.29 (.58-2.90)	2.66 (.68-10.51)
Age	**1.05 (1.03-1.07)**	**1.07 (1.02-1.11)**
Gender, female	1.27 (.68-2.34)	.92 (.36-2.35)
Education, more than 13 years	.83 (.49-1.41)	1.19 (.52-2.69)
Immigration background	.48 (.15-1.56)	Omitted
Sick leave at, or within 1 month, from baseline	**.22 (.08-.63)**	.43 (.09-2.12)
Disability pension before baseline	**2.58 (1.23-5.43)**	.99 (.24-4.09)
Comorbidity, 2 or more	1.88 (.73-4.88)	.33 (.04-3.02)
Body Mass Index	1.03 (.97-1.08)	.95 (.86-1.05)
Clinical prognosis		
Good	Ref	Ref
Medium	1.54 (.83-2.87)	1.74 (.64-4.71)
Poor	1.47 (.72-3.01)	2.93 (.96-8.96)
Diagnosis		
Osteoarthritis	Ref	Ref
Spinal	**.35 (.17-.70)**	**.24 (.06-.89)**
Shoulder	**.23 (.08-.65)**	.55 (.13-2.22)
Knee	**.08 (.01-.62)**	Omitted

Widespread/Fibromyalgia<br>Fracture/injuries<br>Other MSDs	.43 (.16-1.12)<br>Omitted<br>**.10 (.02-.41)**	.54 (.11-2.78)<br>Omitted<br>.29 (.03-2.61)

Table 4: Associations to future disability pension, registered at least 365 days after baseline. Includes patients registered in work the year before baseline, age 63 years or younger and not registered with prior disability pension N=319

	Univariate<br>OR (95% CI)	Multivariate<br>OR (95% CI)
Global Perceived Effect, improved	**.15 (.06-.41)**	**.06 (.01-.53)**
Physiotherapy contacts, median or higher (8+)	1.89 (.98-3.67)	.80 (.14-4.53)
GP contacts, 90th percentile or higher (5+)	3.58 (1.79-7.18)	2.98 (.30-29.34)
Any hospital contact	1.22 (.59-2.50)	.65 (.12-3.56)
Any chiropractor contact		.34 (.02-6.10)
Age	**1.08 (1.04-1.12)**	**1.17 (1.02-1.33)**
Sex, female	**2.75 (1.13-6.67)**	2.52 (.40-15.80)
Education, more than 13 years	**.43 (.23-.84)**	.17 (.03-1.02)
Immigration background	.39 (.09-1.64)	Omitted
Comorbidity, 1 or more	1.49 (.70-3.17)	Omitted
Body Mass Index	**1.10 (1.04-1.16)**	1.07 (.93-1.21)
Clinical prognosis<br>Good<br>Medium<br>Poor	 Ref<br>**6.60 (1.93-22.63)**<br>**13.24 (3.79-46.25)**	 Ref<br>1.83 (.12-27.78)<br>**19.67 (1.27-304.56)**
Diagnosis<br>Osteoarthritis<br>Spinal<br>Shoulder<br>Knee<br>Widespread/Fibromyalgia<br>Fracture/injuries<br>Other MSDs	 Ref<br>1.00 (.38-2.64)<br>.59 (.17-2.10)<br>Omitted (no events)<br>**2.74 (.92-8.14)**<br>2.09 (.56-7.75)<br>.50 (.15-1.63)	 Ref<br>3.57 (.41-31.32)<br>1.07 (.05-21.65)<br>Omitted<br>2.39 (.06-96.43)<br>20.39 (.70-586.97)<br>3.28 (.09-117.36)
